# The Roles of Stroma-Derived Chemokine in Different Stages of Cancer Metastases

**DOI:** 10.3389/fimmu.2020.598532

**Published:** 2020-12-22

**Authors:** Shahid Hussain, Bo Peng, Mathew Cherian, Jonathan W. Song, Dinesh K. Ahirwar, Ramesh K. Ganju

**Affiliations:** ^1^Department of Pathology, The Ohio State University Wexner Medical Center, Columbus, OH, United States; ^2^Division of Medical Oncology, The Ohio State University Wexner Medical Center, Columbus, OH, United States; ^3^Comprehensive Cancer Center, The Ohio State University Wexner Medical Center, Columbus, OH, United States; ^4^Department of Mechanical and Aerospace Engineering, The Ohio State University Wexner Medical Center, Columbus, OH, United States

**Keywords:** chemokines, cancer, metastases, stroma, invasion, metastatic niche

## Abstract

The intricate interplay between malignant cells and host cellular and non-cellular components play crucial role in different stages of tumor development, progression, and metastases. Tumor and stromal cells communicate to each other through receptors such as integrins and secretion of signaling molecules like growth factors, cytokines, chemokines and inflammatory mediators. Chemokines mediated signaling pathways have emerged as major mechanisms underlying multifaceted roles played by host cells during tumor progression. In response to tumor stimuli, host cells-derived chemokines further activates signaling cascades that support the ability of tumor cells to invade surrounding basement membrane and extra-cellular matrix. The host-derived chemokines act on endothelial cells to increase their permeability and facilitate tumor cells intravasation and extravasation. The tumor cells-host neutrophils interaction within the vasculature initiates chemokines driven recruitment of inflammatory cells that protects circulatory tumor cells from immune attack. Chemokines secreted by tumor cells and stromal immune and non-immune cells within the tumor microenvironment enter the circulation and are responsible for formation of a “pre-metastatic niche” like a “soil” in distant organs whereby circulating tumor cells “seed’ and colonize, leading to formation of metastatic foci. Given the importance of host derived chemokines in cancer progression and metastases several drugs like Mogamulizumab, Plerixafor, Repertaxin among others are part of ongoing clinical trial which target chemokines and their receptors against cancer pathogenesis. In this review, we focus on recent advances in understanding the complexity of chemokines network in tumor microenvironment, with an emphasis on chemokines secreted from host cells. We especially summarize the role of host-derived chemokines in different stages of metastases, including invasion, dissemination, migration into the vasculature, and seeding into the pre-metastatic niche. We finally provide a brief description of prospective drugs that target chemokines in different clinical trials against cancer.

## Introduction

One of the key property of cancer cells is the ability to invade the basement membrane, intravasate into the peripheral circulation and colonize in distant organs to establish metastasis nodules, which is the major cause of cancer related deaths (1, 2). For decades, it was believed that cancer cells are self-sufficient to achieve uncontrolled growth and metastases. However, the work done in last few decades strongly support the notion that tumor growth and metastases is rather a complicated process supported by variety of other cell types present in the intimate tumor microenvironment (TME) (3). The process of metastases can be broadly divided into three phases, namely dissemination, spread, and seeding at the site of metastases (4). At the stage of dissemination, tumor cells undergo transcriptional modifications to induce epithelial-to-mesenchymal (EMT) phenotypic change, invade the basement membrane and extracellular matrix (ECM) and intravasate into the vasculature. Once in the vasculature, circulating tumor cells (CTCs) can extravasate at the site of metastases. After extravasating at the site of metastases, CTCs colonize and proliferate to form metastatic foci. Now it is established that intricate interplay between the tumor cells and other cells present in TME participates in all stages of tumor metastases, including invasion, ECM remodeling, intravasation and colonization of distant organs (5).

### Components of Tumor Microenvironment

A tumor mass is a dynamic 3D structure that includes cellular and extracellular components creating a unique TME. The cellular component of the TME is composed of a heterogeneous population of stromal cells (5). These stromal cells are host derived and include innate immune cells, such as monocytes, macrophages, NK cells; adoptive immune cells, including T cells and B cells; and non-immune fibroblast, pericytes, endothelial cells, adipocytes, and mesenchymal stromal cells (MSCs). In addition, ECM and milieu of secreted factors are also integral extracellular components of the TME.

#### Different Mechanisms of Tumor-Stroma Communication

Reciprocal interaction between tumor cells and other cell types occurs in different ways, including direct cell-to-cell contact, secreted molecules and cargo vesicles known as exosomes. Cancer cells express various cell surface ligands that can directly interact with membrane receptors present on other cell types in the vicinity (6). One such class of receptors is the integrins. Integrins bind cells to the ECM and respond to shear stress (6, 7). Cancer cells also shed vesicles loaded with nucleic acids, peptides and metabolites that can fuse with other cells. Extracellular vesicles play role in angiogenesis (8) immunosuppression (9), aid in crosstalk of cancer cells with fibroblasts (10) and development of premetastatic niche (11, 12). A number of different chemokines such as CCR8, CCL18 in glioblastoma (13), CCL2, CCL3, CCL4, CCL5, CCL20 in lung carcinoma have been reported that are packaged and shedded by chemokines in the TME (14). However, the most extensively studied mode of cancer cell interactions is through secreted molecules. The major type of secreted signaling molecules are growth factors, cytokines/chemokines, inflammatory mediators, and metabolites. Soluble ligands secreted by cancer cells bind to their cognate cell surface receptors present on stromal cells, or vice-versa, and activate specific signaling pathways. Cytokines are small protein (5–20 kDa) involved in activating cell signaling by binding to specific cell surface receptor and regulate immunity, inflammation and hematopoiesis. Chemokines are smaller (8–14 kDa) cytokines that are predominantly involved in cell chemotaxis and trafficking. Chemokines have broadly been divided into two subfamilies on the bases of presence (CXC) or absence (CC) of an amino acid between N terminal first two cysteine residues (15). In this review we will discuss the role of chemokines secreted by stromal cells that favor metastases of cancer cells.

#### Innate Immune Cells

Innate cells are one of the major type of cells recruited to the TME. Tumors are known to educate these inflammatory cells to support tumor growth and metastases (16). The major type of inflammatory immune cells in the TME is tumor-associated macrophages (TAMs) and myeloid-derived suppressor cells (MDSCs) (17–20). Classically activated macrophages are the first line of immune defense and are known to clear pathogens from site of infection. However, tumor educated TAMs are incapable of clearing tumor cells due to reduced phagocytic activity. Rather than activating immune response, TAMs suppress immune cells by various mechanisms, including upregulation of checkpoint molecules, secreting immune suppressive molecules like IL-10, TGF-β and prostaglandin-E2 (PGE2) and deviating immune helper cells maturation towards immune suppressive phenotypes (21–23). In addition, TAMs provide growth factors to proliferative cancer cells, secrete ECM degrading enzymes to enhance invasion and escort cancer cells to the vascular interface (24–26). Another type of innate immune cell, MDSCs are immature myeloid cells that are highly immune-suppressive in nature. By metabolizing L-arginine, MDSCs deprive T-cells of a critical substrate for nitric oxide production and inhibit their proliferation and activation. The MDSCs also divert T-cell maturation towards immune suppressive T regulatory (Tregs) cells (27). The MDSCs have broadly been divided into Ly6G-positive granulocytic MDSCs and Ly6C-positive monocytic MDSCs. Monocytic MDSCs suppress both antigen specific and antigen non-specific T cell responses using both cytokines and NO to perform these functions while granulocytic MDSCs inhibit only antigen specific T cell responses [reviewed by (28, 29)]. Monocytic MDSCs impair interferon-α production by increasing STAT1 phosphorylation leading to loss of T cell function and immunosuppression (30). Granulocytic MDSCs produce ROS and inhibit T cell proliferation by downregulation of TCRζ expression, inhibition of NF-κB activation, and cell death by induction of apoptosis (29).

#### Adaptive Immune Cells

Adaptive immune cells can elicit a highly specific immune response against foreign antigens, including cancer. The major types of adaptive immune cells include CD4+ T helper cells, CD8+ T effector cells and B cells. CD8+ T cells are key factor in anti-tumor immune surveillance. The interaction of tumor-antigen presenting dendritic cells and CD4+ T helper cells releases chemokine CCL3 and CCL4 that in turn attract CCR5+ cytotoxic CD8+ T cells (31). Previous studies showed that cancer cells, along with tumor-educated inflammatory cells suppress T-cells to escape immune surveillance. Under the influence of immune suppressive TME, CD4+ T helper cells differentiate from immune promoting phenotype to immune suppressive Treg phenotype (5, 27, 32). In addition, tumor educated B cells coverts CD4+ T cells to immunosuppressive Tregs (33).

#### Non-Immune Cells

Cancer associated fibroblasts (CAF) are the major type of stromal cells that comprise the TME. The contribution of fibroblasts to tumor growth was first inferred in1986, when Picard and colleagues demonstrated the importance of injecting fibroblasts with cancer cells for achieving successful tumor engraftment *in vivo* (34). Later, it was observed that not only direct cell-cell contact but secreted factors are also involved in epithelial/cancer cell-fibroblast interactions (35, 36). Thus, fibroblast-associated tumor-promoting properties have now largely been attributed to growth factors, chemokines/cytokines and metabolites, together known as fibroblast secretome. However, exchange of metabolites and activation of signaling pathways between fibroblasts and tumor cells *via* direct cell-cell contact mechanisms continue to be viewed as important (17, 37). The studies done in the last two decades with transgenic mouse models on oncogenic or antitumor genes have provided strong evidence regarding the role of fibroblasts in supporting tumor cell proliferation, ECM remodeling, metastases and elevating the process of angiogenesis, as reviewed elsewhere (38–40).

Endothelial cells that are recruited to the TME promote neo-angiogenesis. The expansion of blood vasculature ensures adequate perfusion to support overwhelming tumor growth and provides for a route for hematogenous dissemination. The interaction of tumor cells with endothelial cells is a critical step in the process of intravasation, extravasation and metastases whereby cancer cells manipulate pericytes to alter blood vessel integrity and facilitate intravasation (41–43).

#### Extracellular Components

ECM and secreted factors play a dynamic role in tumor biology. The expression of ECM proteolytic enzymes, matrix metalloproteinases (MMPs), is closely associated with tumor progression (44–46). The tumor-induced deposition of ECM at the site of primary as well as metastatic tumor is responsible for chemotherapy and immunotherapy resistance by limiting the entry of drugs to the core of tumor (47).

## Role of Stroma-Derived Chemokines in The Local Invasion of Primary Tumor

**Figure 1:**
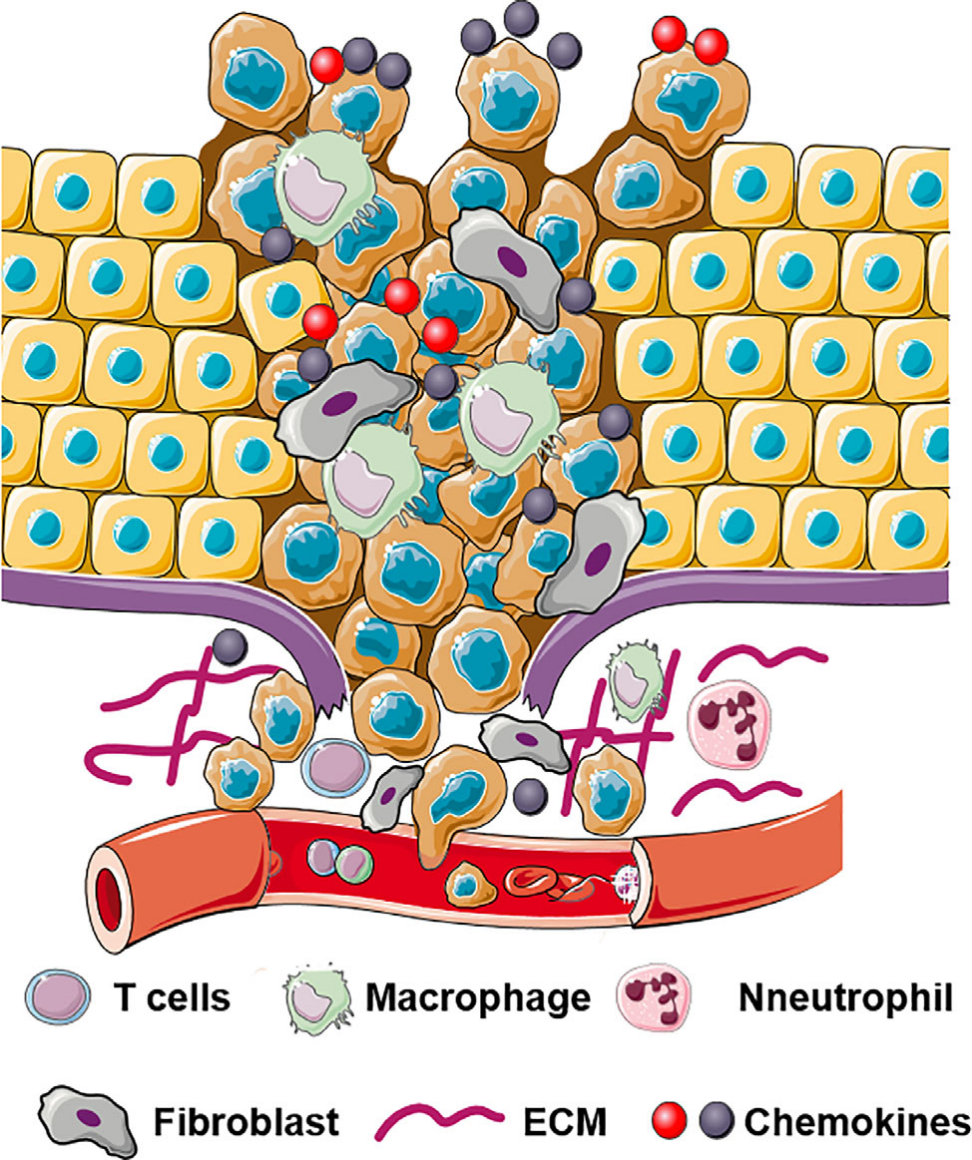
Schematic illustration of multifaceted roles of chemokines in invasion and dissemination. Chemokines bind to their receptors and regulate ECM remodeling, EMT, cell migration, and cell invasion.

The local dissemination of primary tumor cells into the adjacent normal tissues is an initial step of tumor metastases. The sequence of this program involves epithelial-mesenchymal transition (EMT); acquisition of tumor-initiating capability known as cancer stem cell (CSC) properties; cell adhesion to extracellular matrix (ECM) or vascular endothelial cells; extracellular matrix (ECM) remodeling, and cell migration/invasion (48, 49) (**Figure 1**).

Chemokines, play an essential role in the dissemination of cancer cells into adjacent normal tissues. Interactions between tumor and stromal cells promote chemokine production in stromal cells which in turn directly or indirectly stimulate cancer metastases (50). **Table 1** describes multifaceted roles of stromal cells derived chemokines in local invasion by primary tumor.

**Table 1:** Stromal chemokines contributing to local invasion of primary tumor.

Ligands	Receptors	Stromal origin	Type of cancer	Function	Reference
CCL2	CCR2	Fibroblasts, TAM	Hepatocellular carcinoma, prostate cancer, breast cancer, colon cancer, chondrosarcoma	MMP2 secretion, invasion, CSC-like properties	(51–53)
CCL5	CCR1, CCR5	NK cells, T cells, TAM, CAFs, platelets	Breast cancer, prostate cancer, ovarian cancer	MMP2 and MMP9 secretion, EMT, self-renewal of CSCs	(54, 55)
CCL7	CCR1, CCR2, CCR3	CAFs, Adipocytes, Neutrophils	Breast cancer, lung cancer, colon cancer, oral cancer	ECM degradation, invadopodia formation, migration, invasion	(56)
CCL17, CCL22	CCR4	TAMs, CAFs	Prostate cancer, colorectal cancer, hepatocellular carcinoma	EMT, MMP2 expression, migration, invasion	(24, 57–59)
CCL18	CCR8	TAMs, Bone marrow-derived fibrocytes	Lung cancer, oral squamous cell carcinoma, squamous cell carcinoma of the head and neck, endometrial cancer cells	EMT, cytoskeleton reorganization, invasion CSC-like properties,	(60–63)
CCL20	CCR6	TAMs	Hepatocellular, Esophageal squamous cell, breast, gastric, laryngeal carcinoma	EMT, MMP2 secretion, migration, invasion	(20, 64–65)
CXCL1, CXCL5	CXCR1, CXCR2	TAMs, CAFs, endothelial cells	Gastric, prostate, colorectal, lung, bladder, nasopharyngeal, papillary thyroid cancers	EMT, MMP2 and MMP9 secretion, migration, invasion	(26, 66–71)
CXCL8	CXCR1, CXCR2	Endothelial cells, CAFs, TAMs, neutrophils	Breast cancer, pancreatic cancer, gastric cancer, renal cell carcinoma	EMT, invasion, migration, CSC-like properties,	(25, 72–75)
CXCL12	CXCR4 CXCR7 (ACKR3)	Cancer-associated fibroblasts	Breast cancer, ovarian cancer, colorectal cancer, hepatocellular cancer, pancreatic cancer, prostate cancer, thyroid cancer, glioma	cell adhesion, cytoskeletal reorganization, formation and 71, maturation of invadopodia, ECM degradation, CSC, EMT	(18, 44, 76–85)
CXCL16	CXCR6	MSCs, CAFs	Gastric cancer, breast cancer, prostate cancer	adhesion to endothelial cells, cell migration, cell invasion	(46, 86–88)

### Stromal Chemokines and EMT

EMT re-programming is one essential step enabling the invasion and metastatic dissemination of cancer cells. During this process, cancer cells are endowed with malignant traits associated with the loss of epithelial traits and the acquisition of a more mesenchymal phenotype (89). It has been widely accepted that heterotypic signaling pathways induced by chemokines from the tumor-associated stroma can trigger EMT in a variety of carcinomas (49). These have been proven by several co-culture experiments involving cancer cells and host cells such as fibroblasts. CXCL12 expressed by CAFs causes EMT in breast cancer and human tongue squamous cell carcinoma (76, 90). EMT mechanistically was studied in breast (91, 92), thyroid (93)and colon cancer (77). Signaling pathways perturbed were PI3K/AKT/PKB), MAPK/ERK (77, 92), WNT/β-catenin (91) and NF-κB pathways (93). Similarly, fibroblasts when co-cultured with prostate cancer cells produce CXCL1 (which binds to CXCR1 receptor on tumor cells). CXCR1 receptor is also present on neutrophils which produce LCN2. The CXCL1-LCN2 paracrine axis activates Src signaling and leads to EMT and contributes to tumor progression (66). Furthermore, in pancreatic cancer CXCL8 causes cell invasion and promotes metastases (72). Similarly in gastric cancer, neutrophils like cells expressed CXCL8 and induced EMT through CXCR1/CXCR2 receptors (25, 73, 94) and CXCL16 produced by MSCs induces proliferation and migration of tumor cells (86). CAF-derived CXCL16 could also promote brain metastases in breast cancer, which were significantly inhibited by the CXCL16 neutralizing antibody (87).

Several CC chemokines CCL5 and CCL18 also promote EMT, cell migration and invasion in co-culture experiments involving TAMs and different cancer cells. Signaling pathways activated by CC chemokines include NF‑κB and β-catenin/STAT signaling in breast and prostate cancer and MEK/ERK/NF-κB/integrin αvβ3 pathways in osteosarcoma by CCL5 (54), PI3K/AKT/mTOR, and ERK1/2 signaling in endometrial carcinoma and squamous cell carcinoma of the head and neck by CCL18 (60, 95). Furthermore, co-culture of monocyte-derived macrophages which is a major source of CCL20 with hepatoma cells induces EMT and accelerates tumor metastases in a CCL20-dependent manner (20, 61, 64) by activation of P38 MAPK (96), CrkL-ERK1/2 (97) and JAK2/STAT3 pathways (65). In addition, fibroblasts can secret the CC family chemokine CCL17/CCL22 that activates CCR4 and ERK/AKT signaling (57) and plays a critical role in the malignant progression of prostate, breast and hepatocellular cancer (24, 57, 58).

### Stromal Chemokines and CSC-Like Traits

Induction of EMT triggers in several types of cancer cells to acquire cancer stem cell (CSCs) properties such as self-renewal, tumor-initiating and multipotent differentiation potential that enhances metastases (98). Disseminated cancer cells are believed to be endowed with CSC-like properties, which permits mesenchymal differentiation and increased capacity for establishment of metastatic colonies (49). Many studies with CXCL12, CXCL8, CCL2 and CCL18 chemokines have reported a correlation between the stromal chemokines and the acquisition of CSC-like traits (99). In addition, CCL2 was shown to activate the STAT3 and NOTCH1 pathways (51) and macrophages-derived chemokine CXCL8 increases CSCs-like populations and enhances mammosphere formation *via* activation of AKT/mTOR signaling in renal and breast cancer (25, 74).

### Stromal Chemokines and Extracellular Matrix Remodeling in TME

The EMT process confers the polarized epithelial cells properties that are critical to the invasion-metastases cascade, which includes interaction with basement membrane surface receptors and degradation of ECM (49). Stromal CXC and CC chemokine families have been reported to trigger protease release, leading to ECM degradation and play essential roles in cancer metastases (48).

CAF-derived CXCL12 upregulates tumoral expression of matrix metalloproteinases (MMP-2, 9, 13) and thereby leads to contraction of collagen matrices (18, 44, 78). CXCL12/CXCR4 axis also induces the formation and maturation of invadopodia, which alters cellular morphology, induces ECM degradation and promotes invasive features (79) through RhoA/ROCK/MLC-2, Src-Arg-cortactin and MAPK signaling pathways (18, 79). In addition, co-culture of gastric cancer cells and tumor-associated lymphatic endothelial cells (LECs) elevated CXCL1 secretion in LECs which in turn upregulated integrin β1 and MMP2/9 that promotes cell adhesion, migration, invasion and lymph node metastases (67).

Aberrant expression of CC chemokine receptors has also been also related to ECM remodeling processes (48, 56). Chemokine CCL2 and CCL5 in prostate and ovarian cancer promoted MMP-2 and MMP-9 secretion by ERK, Rac signaling (55); in chondrosarcoma through Ras/Raf-1/MEK/ERK/NF-κB and in breast and liver cancer *via* PI3K/Akt and GSK-3β pathways (52, 53). CCL7 interacts with CCR3 and activates RhoA/ERK and PI3K pathways, resulting in collagen degradation and invadopodia formation, contributing to cell invasion (56). CCL17/22 enhanced MMP2 expression *via* ERK/AKT signaling in hepatocellular cancer (57), and upregulate MMP13 expression *via* ERK/NF-κB signaling in colorectal cancer (59). Similarly, CCL20/CCR6 binding leads to MMP2 upregulation *via* JAK2/STAT3 and CrkL-Erk1/2 pathways, and MMP9 upregulation through the activation of PKC-α, src, Akt, and NF-κB pathways in breast and gastric cancer (65, 97).

### Role of Stromal Cell Chemokines in Cancer Cell Dissemination

As discussed above, stromal chemokines act on tumor cells in epithelial cancers and trigger EMT. Subsequently, epithelial cancer cells regulate cell-cell adhesion structures and cell polarity that contribute to cancer cell dissemination. Within invasive carcinomas, the multicellular microanatomical structures called Tumor MicroEnvironment of Metastases (TMEM) serve as the functional sites of tumor cell dissemination and transendothelial migration. Migratory breast, pancreatic, lung and colon cancer cells at TMEM overexpress an invasive form of Enabled (Ena)/vasodilator-stimulated phosphoprotein (VASP) protein (MENA^INV^) in their non-invasive compartments with an intact basement membrane (100) and promotes cancer invasion and migration of tumor cells (101, 102). MENA^INV^ expressing invasive cancer cells enhance transendothelial migration in response to cancer cell-macrophage contact (100, 102). These cells migrate along collagen fibers and stream toward vasculature paired with TAMs driving invadopodium assembly and results in matrix degradation, discohesive tumor morphology and increased tumor cell motility (103, 104). The TAM derived growth factors help cancer cells to form invadopodia and acquire invasive properties *via* EGF/CSF1 paracrine loop and through the activation of Notch signaling (100, 102).

CAF-derived CXCL14 can also modulate cell adhesion and promote motility. CXCL14 interacts with atypical receptor ACKR3 with greater binding affinity and activates Akt and ERK1/2 MAPK by β-arrestin binding (80). Stromal CXCL16 modified cellular adhesion as well as motility by promoting Ezrin activation, αvβ3 integrin clustering and F-actin stability *via* FAK/PI3K/PKC and CXCR6/ERK1/2/RhoA/cofilin/F-actin pathway (46, 88).

## Role of Stroma-Derived Chemokines in Intravasation and Transport of Tumor Cells

The major cause of cancer related death is due to distant metastases. Invasion of basement membrane, ECM and directional migration towards the vasculature is not sufficient for successfully metastases. Cancer cells must migrate across the vessel wall into the blood or lymph vasculature, a process known as tumor cell intravasation. It is a rate-limiting step of metastases and determines the number of circulatory tumor cells (CTCs), which in turn, dictates the probability of metastases. Cancer cells can access the vasculature through passive, active or assisted mechanisms of intravasation. Passive mechanism involves tumor cells getting entrapped in endothelial emboli while active mechanism involves intravasation involves transendothelial migration of tumor cells. In assisted intravasation, TAMs escort tumor cells across endothelium. At the site of metastases, tumor cells again migrate across endothelial cells, a process known as extravasation, to colonize and generate metastatic foci. In this section, we discuss the role of stromal cell-secreted factors, especially chemokines, in the process of intravasation and extravasation.

### Passive Intravasation and Stromal Chemokines

A tumor embolus is a nest of tumor cells embedded in endothelium. A developing, immature or a dilated blood vessel may allow formation of emboli (41, 43). Tumor cells along with tumor stroma has been observed in an embolus (105). Histologic evaluation of extrahepatic bile duct carcinomas showed that the presence of fibroblasts in the tumor embolus positively associates with increased metastases (105). The notion of stromal contribution to emboli-mediated metastases was established with the observation that injection of emboli containing tumor cells and stromal cells increases chances of experimental metastases (42). Very recently, it has been observed that co-injection of tumor cells with CAFs frequently generated emboli with proliferative tumor cells compared to control fibroblasts or no fibroblasts (106) and increases the viability of tumor cells in the emboli (107). Stromal fibroblasts-derived chemokine CXCL12 and Transforming Growth Factor β1 (TGFβ1) induce Src-mediated EMT and proliferate tumor cells present in emboli. Specific inhibition of CXCL12 and TGFβ1 in CAFs inhibited the proliferating tumor cells in the emboli (106) highlighting the importance of CAFs in protecting tumor cells in the emboli.

### Active Intravasation and Stromal Chemokines

**Figure 2:**
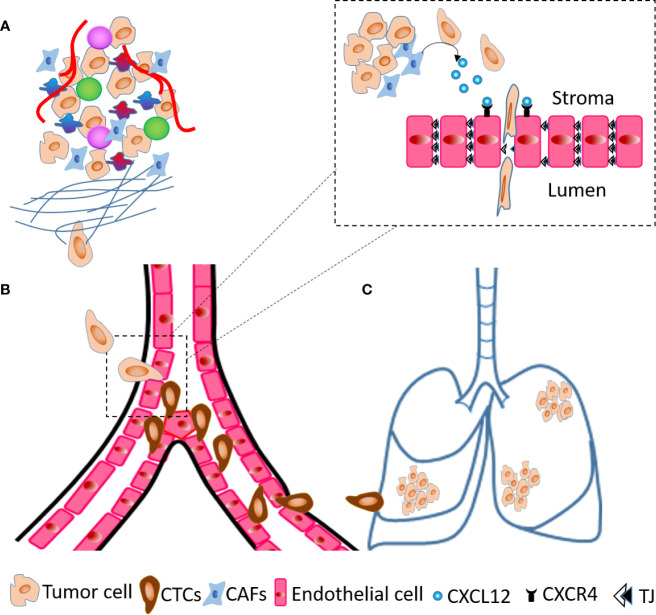
Steps of metastases. **(A)** A cartoon of TME showing tumor cells invade ECM and migrate towards blood vessels. **(B)** At the blood vessels, tumor cells transmigrate across endothelial layer and become circulatory tumor cells (CTCs). **(C)** Tumor cells extravasate and seed into the lungs to generate metastatic foci. The dashed line inset diagram describes molecular events occurring at the time of transendothelial migration shown in **(B)** within dashed line box. The CAFs present in TME secrete CXCL12 that act on endothelial cells through receptor CXCR4 and decrease the expression of tight junction (TJ) molecules, which results in intravasation of tumor cells.

As an active mechanism, tumor cells degrade the basement membrane and intravasate through trans-endothelial migration. During this process, tumor cells undergo EMT, achieve invasive and highly motile phenotype and secrete factors to increase permeability of blood vessels in the TME. CXCL12 expressing organs are a target for CXCR4 positive cancer cells and blocking the CXCL12/CXCR4 interaction using neutralizing antibodies (nAb) significantly inhibits experimental metastases (108). We and others have established fibroblasts as a critical source of CXCL12 in the TME (3, 40, 109). Co-implantation of human CAFs secreting CXCL12 with tumor cells enhances breast tumor growth (40) with CXCL12 recruiting endothelial cells to enhance angiogenesis. We have shown that, depletion of fibroblast-specific CXCL12 results in reduced tumor growth, decreased number of CTCs and inhibits metastases of orthotopic and spontaneous mammary tumors (109). Fibroblast-derived CXCL12 expands the leaky tumor vasculature by recruiting endothelial precursor cells and suppressing tight junction molecules and this facilitates tumor cell intravasation (109). Using RNA hybridization technique, we confirmed that fibroblasts are a critical source of CXCL12 and that fibroblast specific deletion of CXCL12 abrogates CXCL12 protein expression in the TME suggesting that stromal fibroblasts-secreted chemokine CXCL12 shapes the TME in favor of tumor growth and metastases. The major steps of metastases, especially the role of fibroblasts-derived CXCL12 in intravasation are summarized in **Figure 2**. Besides CXCL12, stromal fibroblast and mesenchymal stem cells (MSCs) also indirectly upregulate chemokines such as CXCL8 and CCL2 through inflammatory molecules like TNF-α and IL-1β (109–111). Importantly, TNF-α stimulated cancer cell-MSC or cancer cell-fibroblast co-cultures stimulated endothelial cell migration and sprouting *in vitro* and increased tumor growth *in vivo* (110). In another study, co-culture of tumor cells and MSCs elevated CCL5 expression in MSCs which increased invasive and migratory properties of tumor cells that express its receptor, CCR5, leading to increased metastases (112).

### Role of Stromal Chemokines in Assisted Intravasation

The passive and active mechanisms of intravasation assume a tumor vasculature that is disorganized and hyper-permeable and thus conducive for tumor cells intravasation. However, researchers suggest that tumor cells are assisted by macrophages during the process of intravasation. A direct association of tumor cells and macrophages during migration was demonstrated by Wyckoff and colleague in 2004 using intravital imaging. Another study using multiphoton *in vivo* imaging showed that perivascular macrophages directly interact with tumor cells and endothelial cells of blood vessels creating the TMEM. TMEM density directly correlates with systemic metastases (32).Within the TMEM, macrophages promote tumor cell intravasation (113). *In-vivo* imaging has shown that tumor cell intravasation occurs only at TMEM doorways and tumor cells are escorted by Tie2 positive TAMs to blood vessels (114). Tie2-positive macrophages locally and transiently compromise vascular integrity by expressing VEGF-allowing intravasation of tumor cells by compromising endothelial cell-cell junctions. Others have suggested that VEGF-A-induce increases in endothelial permeability is a transient and regulated phenomenon (115). Although the role of chemokines secreted by Tie2-positive macrophages has not been studied in TMEM, these cells are known to express chemokines in rheumatoid arthritis (116). In addition, fibroblast-derived CXCL12 can also recruit CXCR4+ TAMs which can alter vessel permeability to facilitate intravasation (117).

The mechanistic studies showed existence of an active EGF/EGFR and CSF1/CSF1R feedback loop between tumor cells and macrophages in renal clear cell carcinoma, glioblastoma and breast cancer (118–122). ErbB3 overexpressing MTLn3 breast cancer cells are more invasive than normal MTLn3 cells in presence of ErbB3 ligand HRG-β1. MTLn3-ErbB3 and transgenic MMTV-Neu tumors invasiveness in presence of HRG-β1 is inhibited by blocking EGFR, CSF-1R, or macrophage function, indicating that invasiveness to HRG-β1 is dependent upon the EGF/CSF-1 paracrine loop. Furthermore, CXCL12 also triggers *in vivo* invasion of transgenic MMTV-PyMT tumors in an EGF/CSF-1–dependent manner (Hernandez et al., 2009). Similarly, co-expression of CSF-1 and its receptor CSF-1R on renal tubular epithelial cells (TEC) promotes proliferation and inhibits apoptosis during regeneration of renal tubules. When CSF-1 and CSF-1R are coexpressed in renal cell carcinoma and TEC aids in RCC survival and inhibition of apoptosis (122).

## Role of Stromal Chemokines in Survival of CTCs in Circulation

Once in the vasculature, cancer cells face various barriers such as physical stress, oxidative stress, anoikis, and the lack of growth factors and cytokines to survive and successfully extravasate and colonize in distant organs. Millions of tumor cells are shed from primary tumor into the efferent blood of mammary adenocarcinoma (123). However, only about 0.1% cells survival and the rest become non-viable within 48 h (124). There are several chemokine-mediated mechanisms that are involved in survival or killing of CTCs. One of the mechanism of CTC death is through immune cell derived chemokine/chemokine receptors. It has been shown that CX3CR1^+^ monocytes are recruited to the tumor cell aggregates in response to tumor-derived CX3CL1, where they can directly engage cancer cells or secrete CCL3, CCL4 and CCL5 to activate natural killer cells to kill CTCs (125). CCL2 is a potent chemoattractant for phagocytic cells including monocytes, and macrophages (126).

Tumor cells secrete thrombin to activate platelets to aggregate around tumor cells. The activated platelets cross-link with each other to create tumor cell-platelet microemboli. In addition, tumor cell-platelet association also inhibits NK cells mediated lysis of CTCs (127, 128). In addition to protecting CTCs in the vasculature, platelets are known to secrete CXCL12, which promotes their survival, transendothelial migration and extravasation (129, 130). As another mechanism to support CTCs survival, platelets secrete lysophophatidic acid (LPA) that binds to its receptors, LPAR1, 2 and (3) on breast cancer cells and activates the secretion of IL6, IL8, CCL2 and CXCL1, enhance survival and increases the migration potential of CTCs (131).

### Role of Stromal Chemokines in Establishing Pre-Metastatic Niche

Tumor cells in distant organs induce formation of a microenvironment that promotes the establishment and growth of tumors and this was subsequently referred to as the ‘pre-metastatic niche’ (PMN) (132–134). PMNs arise as a result of combined efforts of tumor secreted factors, stroma secreted factors and tumor-shed extracellular vesicles (EVs) that regulate the stepwise evolution of PMN (133). Establishing a PMN involves changes at the local and systemic levels. Locally, within the PMN, changes arise in the stroma, vasculature and ECM (135, 136).

The expression of CXCR4 receptor on tumor cells aids in their colonization to organs expressing CXCL12 such as bone marrow which favors tumor cell survival and growth (137). Activation of CXCR4 receptor by CXCL12 has been reported in different cancers such as brain neoplasms, colorectal cancer, prostrate cancer, melanoma, ovarian cancer, etc (138–142). SCLC overexpresses CXCR4 and activation by CXCL12 overexpressed in bone marrow induces migration, invasiveness and adhesion to marrow stromal cells which prevent apoptosis in SCLC cells from chemotherapy (137, 143, 144).

Formation of PMNs in lungs is reported to be initiated by accumulation of VEGFR1^+^ bone marrow derived hematopoietic progenitor cells along with resident fibroblasts which express high levels of CXCL12. VLA4 is expressed by VEGFR1+ cells which causes resident stromal fibroblasts to upregulate fibronectin which forms a permissive niche for incoming tumor cells (132). The hematopoietic progenitor cells promote migration and adhesion of lung carcinoma and CXCR4^+^ B16 melanoma cells to the lung PMNs (132). Furthermore, lung epithelial cells secrete chemokines such as CXCL1, CXCL2, CXCL5 and CXCL12 to recruit neutrophils in Lewis Lung Carcinoma (LLC) xenograft models in response to TLR3 activation by LLC exosomal RNAs (145, 146). In the liver, CXCL12 is involved in PMN formation through CXCL12-CXCR4 dependent recruitment of neutrophils that enhance PMN properties by secretion of chemokines such as tissue inhibitor of metallopeptidase 1 (TIMP1) (111, 147, 148). In the lymph nodes, CCR7^+^ melanomas colonize and inhibiting the CCL21 with neutralizing antibodies blocks CCR7 mediated metastases (149). In the central nervous system, CCR7 expression in the brain endothelial cells, is an essential adhesion signal for CCR7+ leukemia T-cells to specifically seed and metastasize to central nervous system. Silencing of the CCR7 or its ligand CCL19 in animals models has been reported to inhibit infiltration of leukemia cells into the brain (150).

Besides the stromal changes, one of the major vascular changes which prime distant tissue for metastatic cell colonization is formation of blood clots due to leaky vasculature (151). Vascular clots rich in fibrin and platelets coat the surface of CTCs and aid in their dissemination (152). Platelets secrete CXCL5 and CXCL7, contact with tumor cells cause recruitment of granulocytes (CD11b^+^MMP9^+^Ly6G^+^) to tumor cells in lung to form early metastatic niches directly helping in tumor cell seeding and development of lung metastases (153, 154). In addition, CTCs homing and survival in PMNs is aided by tissue factor mediated clot formation in which macrophage populations are already recruited during PMN formation (155).

### Role of Stromal Chemokines in Extravasation of CTCs at PMNs

After surviving the hostile vascular environment, CTCs undergo trans-endothelial migration to extravasate from the blood vessel into local PMN. The host cells co-ordinate with each other in response to tumor signals to help extravasation of CTC. Upon activation by tumor cells, platelets secrete CXCL5 and CXCL7 chemokines to recruit CXCR2-positive neutrophils (153). These neutrophils release cytosolic and granulated proteins that are assembled along with chromatin fibers to generate large, web-like structures know as neutrophil extracellular traps (NETs) (153, 156). NETs have been reported in pancreatic, liver, breast and gastric cancers (157–160). In metastatic breast cancer, neutrophils form metastatic supporting NETs. NET-DNA acts as a chemotactic signal to attract cancer cells rather than just acting as a trap at distant metastatic sites (161). These NETs contributes to the extravasation by sequestering CTCs and degrading the ECM to facilitate migration of CTCs (156, 162). In addition, tumor-derived CCL2 can directly act on CCR2 positive endothelial cells to increase their permeability or indirectly *via* increasing CXCL12 expression, which helps CXCR4 positive cancer cells to extravasate into the PMNs (129, 163, 164). Finally, the CCL2 from tumor cells and CCL5 from activated endothelial cells can further recruit the inflammatory monocytes/macrophages to prepare the cellular assembly of CTCs, platelets, neutrophils, endothelial cells and monocytes/macrophages required for efficient extravasation of CTCs (154). Activation of CCR2 signaling prompts TAMs to secrete another chemokine, CCL3, leading to enhanced TAMs–tumor cell interaction and prolonged retention of TAMs in the metastases sites, which promotes extravasation of breast cancer cells (165, 166).

### Role of Chemokines in Establishing Metastatic Foci

**Figure 3:**
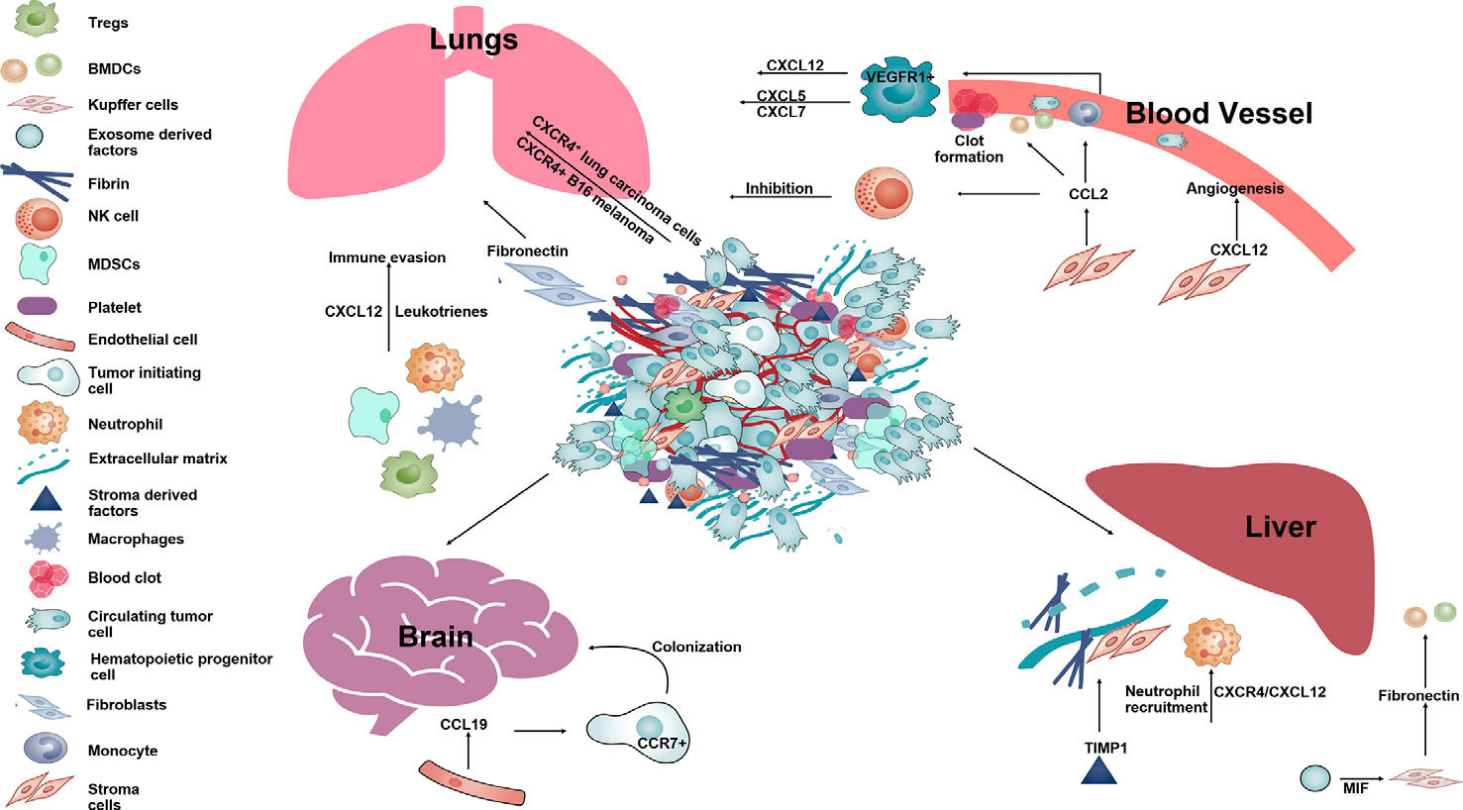
The schematic diagram showing different function of stromal-derived chemokines in pre-metastatic niche formation and metastases. Tumor cells along with stromal and immune cells in the primary tumor secrete several chemokines such as CCL2, CXCL12, CCL19, etc., that help in establishing the PMN in distant organs such as lung, liver, brain. Within the lungs SDF-1 and leukotrienes help in immune invasion. CCL2, CXCL12 aid in angiogenesis, form clots in blood vessels and help in survival of melanoma in the lung by inhibiting NK cell activity. In the brain, CCL19 acts on CCR7+ tumor cells and aid in their colonization. In the liver, CXCL12 along with TIMP1 and MIF help in neutrophil recruitment and deposition of fibronectin making liver tissue permissive for formation of a metastatic niche for circulating tumor cells.

As final step of metastases, tumor cells have to seed, survive, and proliferate into the PMC to develop metastatic foci. CCR2+ inflammatory monocytes recruit to the PMN and are differentiate into metastases associated macrophages (MAMs) (165). These MAMs express another cytokine CCL3 to help seeding of CTCs as it has been shown that the genetic deletion of CCL3 or CCR2 prevents metastatic seeding of breast CTCs (166). High expression levels of chemokine CXCL8 and CCL2 in the bone marrow microenvironment promotes the survival of leukemia cells by enhancing their adhesion to bone marrow MSCs (167). In melanoma too, CCR2+ BMDCs inhibit proliferation of T cells and help in immune escape (168, 169). Regulatory cells such as MDSCs, macrophages, Tregs in the PMNs are also responsible for suppressing anti-tumor responses and help in tumor cell survival (170, 171). MDSCs cause immune suppression at PMNs resulting in tumor cell survival and promote metastases by downregulation of IFNγ which causes expression of pro-inflammatory cytokines, interleukins and CXCL12 (147, 148, 172). Mechanistically, MDSCs inhibit activity of T cells through arginase1 and reactive oxygen species (ROS) production (27, 173). Tumor evoked B regulatory cells (tBregs) play a primary role in lung metastases by the conversion of resting anti-tumor CD4+ T cells into FoxP3+ expressing Treg cells through the paracrine action of TGFβ and CCL22 expressed from lung (33). The studies discussed above describe the functions performed by stromal cells-derived chemokines throughout the multi-step process of metastases (**Figure 3**).

## Therapeutic Targeting of Chemokines

Given that chemokines and their receptors have been recognized as the key regulators of cancer progression, strategies targeting different chemokine/chemokine receptor axes exert antitumoral and antimetastatic activities in many tumors to counteract cancer growth and dissemination, inhibiting angiogenesis and regulating the leukocyte recruitment (174, 175).

CXCR4 inhibitors have been reported promising therapeutic effects on solid tumors, including glioblastoma, breast cancer and mesothelioma. A phase I trial (NCT01837095) with CXCR4 antagonist Balixafortide and Eribulin chemotherapy evaluated the safety, tolerability, pharmacokinetics and efficacy in heavily pretreated, relapsed breast cancer patients. The combination has given promising results in HER-negative metastatic breast cancer (176). Current phase I/II study showed that CXCR4 inhibitor Plerixafor was well tolerated as an adjunct to chemoirradiation in newly diagnosed glioblastoma patients and reduced tumor local recurrences by inhibiting postirradiation tumor revascularization (177). Very recently it has been shown that CXCR4 inhibitor AMD3100 decreased desmoplasia, immunosuppression and increased T cells infiltration into tumors, and thereby enhanced the efficacy of immune checkpoint inhibition in a pre-clinical murine model of breast cancer (178).

CXCR1/2 inhibitor Repertaxin enhanced chemotherapeutic efficacy of 5-fluorouracil in gastric cancer by attenuating cell proliferation, inducing apoptosis and suppressing malignant behavior (179). In pancreatic adenocarcinoma (PDAC), genetic depletion of CXCR2 prevented neutrophil accumulation and improved T cell entry, which contributed to tumor suppression and therapeutic response of anti-PD-1 (180, 181). Additionally, combination of SB225002 (CXCR2 inhibitor) with RS504393 (CCR2 inhibitor) overcame the compensatory response of myeloid subset, augmented antitumor immunity and improved chemotherapeutic response of FOLFIRINOX in an orthotopic model of pancreatic adenocarcinoma (182). A phase Ib clinical trial (NCT02001974) using combination of CXCR1/2 inhibitor Reparixin with paclitaxel was tolerable and safe and showed positive response in metastatic breast cancer (183). Another preclinical study also showed that the anti-CCR4 antibody, Affi-5, exerted antitumor activity in renal cancer models by regulating Th1/Th2 switch, altering myeloid cell phenotype, increasing NK cells and Th1 cytokine levels and reducing immature myeloid cells infiltration (184).

It has been reported that interference with the CCL2/CCR2 axis shows antitumoral activity for reducing monocytes recruitment (175). However, cessation of antiCCL2 therapy leads to an overshoot of metastasis and accelerates death (185). In murine pancreatic cancer models, CCR2 antagonist combined with anti–PD-1 decreased tumor burden by blocking monocyte/macrophage recruitment and relieving suppression of the CD8+ T cells (186). In an orthotopic murine model of hepatocellular carcinoma, blocking CCL2/CCR2 axis with a natural product from *Abies georgei* named 747 or RDC108 suppressed liver tumor growth and recurrence *via* inhibition of TAMs and other immune suppressive myeloid cells (187, 188). Encouraging results have been reported in other clinical studies. A phase I trial of CCR2 antagonist CCX872 plus chemotherapy FOLFIRINOX has been evaluated in non-resectable pancreatic cancer patients (NCT02345408). Compared to median overall survival of 18 months in FOLFIRINOX only treated patients, the median overall survival was increased by 18.6–29% in CCX872/FOLFIRINOX treated patients. The better overall survival was associated with decreased immune suppression by reducing inflammatory monocytes, circulating monocytes and monocytic myeloid derived suppressor cells (189). A phase Ib study (NCT02732938) evaluated the effect, safety and tolerability of CCR2 inhibitor PF-04136309 plus nab-paclitaxel and gemcitabine in aggressive pancreatic cancer (190). The same inhibitor in combination with FOLFIRINOX chemotherapy has been evaluated in a pancreatic cancer clinical trial (NCT01413022) (191). However, targeting the CCR2 ligand CCL2 has not shown promising results in clinical trials. A clinical trial (NCT00537368) using human CCL2 neutralizing monoclonal antibody CNTO888 could not completely inhibit CCL2 in prostate cancer patients and did not show clinical response compared to standard therapy. Other clinical trials using CNTO888 could not achieve any clinical response in solid tumor patients (NCT01204996) (192). These studies emphasize the importance of thoroughly evaluating different strategies for targeting chemokine- signaling pathways to develop novel therapeutic strategies against difficult-to-manage aggressive malignancies.

## Conclusion

Chemokines are crucial molecules involved in autocrine and paracrine signaling required for proper functioning of multicellular organisms. Dysregulation of chemokine secretion can predispose to significant pathology, including malignancies. The emergence of the field of tumor microenvironment have revolutionized the understanding of how tumors exploit host tissues to successfully proceed through different stages of tumor progression, leading to disseminated metastases. The recent advances in the field of TME has shifted the focus of research from targeting cancer cell- autonomous functions to include the host components for designing novel therapies. As a major mechanism of metastases, the tumor educated host cells secrete chemokines and cytokines that feedback on tumor cells and help in dissemination, hematogenous spread and metastatic colonization. Therefore, hindering chemokine signaling pathways between tumor and host cells as a therapeutic strategy against metastatic tumors is gaining increased attention. Recent findings have also established the critical role played by host cells, especially those of the immune system, in the success of conventional radiation therapy and chemotherapy. Therefore, translational significance of targeting host-derived chemokines should be considered in developing novel therapies against cancer, especially metastatic cancer.

## Author Contributions

SH and BP gathered the information, shortlisted the relevant studies and prepared the layout of review. RG and DA organized different sections of the review, discussed the findings and thoroughly reviewed the manuscript. MC reviewed the section describing clinical studies. JS reviewed the angiogenesis and permeability related sections of the review. All authors contributed to the article and approved the submitted version.

## Funding

The publication was supported in part by NIH grants R01CA109527 and DoD breast cancer breakthrough awards (W81XWH1910088, W81XWH1710025, W81XWH1610037) to (RG).

## Conflict of Interest

The authors declare that the research was conducted in the absence of any commercial or financial relationships that could be construed as a potential conflict of interest.
